# Parental Stress of Preschool Children With Generalized Anxiety or Oppositional Defiant Disorder

**DOI:** 10.3389/fped.2019.00415

**Published:** 2019-10-17

**Authors:** Filippo Manti, Federica Giovannone, Carla Sogos

**Affiliations:** Unit of Child Neurology and Psychiatry, Department of Human Neuroscience, Sapienza University of Rome, Rome, Italy

**Keywords:** parenting, stress, GAD, ODD, preschool children

## Abstract

**Background:** Generalized anxiety and oppositional defiant disorders are among the most common psychopathological disorders in pre-school children. We investigated the symptom rate and perception of the child's disorders in parents of preschool children with generalized anxiety disorder (GAD) or oppositional defiant disorder (ODD).

**Methods:** The parents of preschool children (mean age 54.35 months, SD ± 11.60) with ODD, GAD, or typical development (TD) filled the Symptom Check List-90-Revised (SCL-90-R) and the Child Behavior Checklist- 1½−5 (CBCL 1½ 5). Parents and children's diagnoses were determined by clinical assessment.

**Results:** The parents of children with ODD reported a symptoms rate higher than parents of children with GAD or TD on most of the SCL-90-R (Global Severity Index of mental distress, *p* = 0.010; Somatization, *p* = 0.002; Paranoid Ideation, *p* < 0.000; and Phobic Anxiety scales, *p* = 0.030).

**Conclusions:** On the CBCL scales, the parents of the ODD group overestimated the children's problems, while parents of children with GAD reported mainly children's emotional difficulties. Parents of children with ODD seem to be psychologically more vulnerable than parents of children with GAD. Parenting programs might be tailored considering the child's diagnosis and family functioning.

## Introduction

Oppositional defiant disorder (ODD) and generalized anxiety disorder (GAD) are among the most common psychiatric disorders in pre-school-aged children. The key feature of GAD is excessive and intrusive worrying that disrupts daily functioning and is associated with symptoms of restlessness, fatigue, difficulty concentrating, irritability, muscle aches or tension, or sleep difficulties. Children with ODD presented a persistent pattern of behavioral problems, including anger, irritability, arguing, defiance, or vindictiveness toward parents and other authority figures (1–4).

Early childhood anxiety disorders are predictive of anxiety and mood disorders in later childhood. Luby et al. showed that pre-school depression is not a transient or non-specific developmental phenomenon but an early expression of the same chronic and relapsing disorder during late childhood and adolescence (3, 5, 6). On the other hand, children with ODD may develop more serious conduct disorders and antisocial personality disorders in adulthood (7–10). The emergence of children's problems or the exacerbation of existing disorders is often associated with the exposure to stressful social environments. Early difficulties may persist and worsen throughout late childhood, adolescence, and into adulthood (11, 12), especially when the child receives inadequate psychological support from the primary caregivers (13–19). Identification of environmental factors associated with the development of GAD or ODD in children presents an important area for research.

Within a project aimed at identifying the protective and risk factors for psychopathological trajectories, the present study investigated the parental symptom rate and parental perception of the child's psychiatric disorders in parents of children with ODD, GAD, or typical development (TD).

Family history of psychopathology (psychological problems) in pre-schooler children increases the risk for the development of psychiatric disorders in offspring (20–23). In particular, the severity of parental depression is positively associated with high risk of developing internalizing and externalizing disorders in children (24–28). We therefore explored the symptom rate and the presence of a psychiatric diagnosis in parents.

To measure parents' perception of their own personal mental health conditions, all parents were asked to complete the Symptom Check List-90-Revised (SCL-90-R) (29–31). We expected that parents of children with ODD would report greater psychological distress compared to parents of children with GAD and TD group.

We hypothesized that parents of children with GAD would be less aware of the psychopathology of their children than parents of children with ODD. Unlike children with ODD, children with GAD show behaviors that are often underscored by the parents (32). We expected, therefore, that children with GAD would be referred at older ages than children of the other two groups. In addition, we predicted that, on the total score of the Child Behavior Checklist- 1½−5 (CBCL 1 ½ 5), parents of children with GAD would rate their children below the clinical range more often than parents of children with ODD (33, 34). Parents of children with GAD would also be less accurate in recognizing specific child psychopathological symptoms than parents of children with ODD on the CBCL subscales.

In summary, despite the steady and increasing interests toward psychopathological conditions in childhood, relatively few studies focused on the family environment in pre-school children with GAD and ODD (35–37). The present study is aimed at covering this gap and offering new insight into parental awareness levels of the psychiatric problems of their own children and parental perception of their own personal mental health conditions.

## Materials and Methods

### Participants

The sample consisted of 124 Italian preschool children (80 males, 44 females; mean age = 54.35; SD = 11.60; range 36–72 months) and their parents. The children were recruited over the last 5 years (2013–2018).

The psychiatric disorders were assessed according to DSM-5 criteria and confirmed by the Kiddie Schedule for Affective Disorders and Schizophrenia interview (K-SADS-PL) as well as by a complete clinical assessment (38–40). Preschoolers had GAD (*n* = 41) or ODD (*n* = 41) diagnosis.

The comorbidity with any additional psychiatric disorders (ADHD, specific phobia, selective mutism, and separation anxiety disorder) and/or QI < 80 were considered as exclusion criteria. All children were free of medication at study entry.

A total of 42 children with typical development were recruited through advertisements among nursery school. These children were not impaired on relational and psychological levels.

Patients and children with TD had a comparable gender ratio, age, and group level according to their socio-demographic background (see Table 1).

**Table 1:** Sample demographic characteristics.

**Sociodemographic variables**	**ODD group *n* = 41 M (SD)/Freq (%)**	**GAD group *n* = 41 M (SD)/Freq (%)**	**TD group *n* = 42 M (SD)/Freq (%)**	***p***
Child age (months)	55.24 (9.68)	56.46 (10.75)	51.42 (13.64)	0.118
Male	28 (68%)	25 (61%)	27 (64%)	–
Female	13 (32%)	16 (39%)	15 (36%)	–
Mother age	37.00 (5.13)	35.93 (4.60)	36.80 (4.55)	0.554
Father age (yy/mm)	40.66 (6.08)	39.46 (5.67)	39.80 (5.54)	0.628
Ethnicity				
Caucasian	40 (97.5%)	41 (100%)	40 (95%)	–
Asian	1 (2.5%)	–	2 (5%)	–
Parental psychiatric status				
Maternal psychiatric disorders	6 (15%)	7 (17%)	1 (2%)	–
Paternal psychiatric disorders	7 (17%)	8 (20%)	–	–
Life events	32 (78%)	31 (76%)	11 (26%)	–
Average income family				
Below € 30.000 per year	11 (27%)	9 (22%)	10 (24%)	–
€ 30.000–60.000 per year	22 (54%)	20 (49%)	21 (50%)	–
Above € 60.000 per year	8 (19%)	12 (29%)	11 (26%)	–

*M, Mean; SD, standard deviation; ODD, Oppositional Defiant Disorder; GAD, Generalized Anxiety Disorder; TD, Typical Development*.

### Procedures

All the children and their parents were evaluated by trained clinical psychologists and child psychiatrists.

Clinical assessment of all children and their parents was performed according to a three-step process.

The first step consisted of an anamnesis (family history, demographic data, developmental stages, education, income), clinical interview with the parents together, and psychiatric assessment of the child.

In a second step, the children performed a comprehensive neuropsychiatric evaluation (cognitive, neuropsychological, and psychopathological), during which the disorder was confirmed. Both parents were also asked to assess the behavioral and emotional problems of their children by filling out a symptom checklist together (Child Behavior Check List for 1½−5 years, CBCL 1 ½ 5). The questionnaire regarding the parents' psychological profiles (Symptom Checklist-90-Revised, SCL-90-R) was filled out by the mother and father separately.

In the third step, the clinicians discussed the parents' psychopathological risk and the child's diagnosis with the parents.

The study was carried out in accordance with the Declaration of Helsinki and approved by the institutional review board of “Sapienza” at the University of Rome.

All parents gave a written informed consent to participate in this study.

### Measures

#### Anamnesis

The anamnesis of children was carried out by a child psychiatrist asking specific questions (family history, demographic data, developmental stages, family average income, and education level).

#### Psychiatric Assessment

The psychiatric diagnosis of the children was made according to DSM-5 criteria and confirmed by the K-SADS-PL interview.

The parents' psychiatric diagnosis was made by a psychiatrist from the adult mental health service.

#### Symptom Checklist-90 Revised

The Italian version of the SCL-90-R was used to measure the psychiatric distress of family caregivers for children with ODD, GAD, and TD (31).

The SCL 90-R consists of 90 multidimensional items. Each of the 90 items was rated on a five-point Likert scale (from 0 = “no problem” to 4 = “very serious”). Subsequently, the answers were combined in nine primary symptom dimensions: somatization (SOM), obsessive-compulsive (O-C), interpersonal sensitivity (I-S), depression (DEP), anxiety (ANX), anger-hostility (HOS), phobic anxiety (PHOB), paranoid ideation (PAR), and psychoticism (PSY).

In addition, three global indices provided measures of psychological distress: the Global Severity Index (GSI), the Positive Symptom Total (PST), and the Positive Symptom Distress Index (PSDI). The SCL-90-R comprised seven additional items (Other) to measure possible disorders in appetite and sleep. Following Derogatis (29), we used a T-score of 63 as the cut-off point for severe symptoms. The GSI cut-off score was ≥0.57.

#### Child Behavior CheckList for 1½−5 Years

The CBCL 1½−5 is a parent questionnaire assessing emotional and behavior problems in children. Parents rated their children on 100 items using a 3-point scale: 0 = “not true,” 1 = “somewhat or sometimes true,” or 2 = “very true or often true.” A Total Problem score, two broadband scores (Internalizing and Externalizing Domains), and six different syndrome scales were deployed.

Six syndrome subscales contributed to either the broadband Internalizing or Externalizing domains. Only one syndrome scale, Sleep Problems, did not contribute to either broadband but did contribute to the Total Problems Score.

Syndrome scales: borderline clinical range spanning from T Score 65–69 (93rd to the 97th percentile of the normative sample of non-referred children). Scores above the 97th percentile were considered to be within clinical range.

Internalizing, externalizing, and total scales: borderline range spanning from T score 60–63 (83rd−90th percentile).

Also, the borderline DSM-oriented Scale ranges from T score 65–69 (93rd−97th percentile of the normative sample of non-referred children).

### Statistical Analysis

The analysis of demographic variables was performed by the analysis of variance (ANOVA) for continuous measures (i.e., age) and the Pearson's χ2 for categorical measures (e.g., sex, socio-cultural, and occupational status).

For the SCL-90-R and CBCL 1½−5, differences between groups were analyzed by means of analysis of variance followed by *post-hoc* analysis with an adjustment for “child's age” as covariate. Parents (mother vs. father) were considered as a within-subject factors. Linear and logistic regressions were performed to determine the association between parent and offspring psychopathology. To measure the magnitude of the risk for psychopathology in children, an odds ratio was calculated for children with and without psychopathological parents.

Statistical analyses were performed using SPSS version 23.0 (IBM Corp., Armonk, NY, USA).

## Results

We found no significant differences across groups regarding the parents' age (mean = 38.2 months, SD = 5.4) and education level. Overall, the majority of families (73%) had a middle education level, and the remaining families had either low (9%) or high (18%) education levels (low: primary school; middle: high school; high: university).

In most of the cases (75%), the children were referred for diagnosis by the parents. Only in 9% of cases were children referred by teachers, and in 16% of cases they were referred by pediatricians, and there were no differences between groups.

With respect to the sex and age of the children, there were not significant differences between groups. Across the groups, the majority of children were males (64%).

Overall, 14 mothers and 15 fathers had symptoms of anxiety and/or depression.

We found that children with a psychiatric diagnosis were more likely to have at least one parent with psychopathology than TD children (χ = 11.5, df = 2, *p* = 0.003). As expected, parental psychopathology was associated with a strong likelihood of GAD (odds ratio = 16.5, *p* = 0.010) and ODD in children (odds ratio = 15, *p* = 0.009). However, the presence of a parental psychiatric diagnosis was not associated with the type of referral (e.g., parents, school, or pediatricians) of children.

On the SCL-90-R scales, parents of children with ODD reported higher scores than the other parents on the global severity index of mental distress [GSI: F_(2, 120)_ = 4.5, *p* = 0.010; $\eta_{\text{p}}^{2}$ = 0.1], positive symptom total [PST: *F*_(2, 120)_ = 5, *p* = 0.010; $\eta_{\text{p}}^{2}$ = 0.1], and the positive symptom distress index [PSDI: *F*_(2, 120)_ = 4.3, *p* < 0.010; $\eta_{\text{p}}^{2}$ = 0.2].

On most of the SCL-90-R subscales, parents of children with a psychiatric diagnosis reported higher scores on the Anxiety (GAD) and Hostility (HOS) subscales [GAD: *F*_(2, 120)_ = 4, *p* = 0.020; $\eta_{\text{p}}^{2}$ = 0.3; HOS: *F*_(2, 120)_ = 4.4, *p* = 0.014; $\eta_{\text{p}}^{2}$ = 0.2], compared to the TD group. Furthermore, parents of children with ODD reported higher scores on Somatization (SOM), Paranoid Ideation (PAR), Phobia (PHOB), and sleep and appetite disorders compared to the other groups [SOM: *F*_(2, 120)_ = 4, *p* = 0.002; $\eta_{\text{p}}^{2}$ = 0.2; PAR: *F*_(2, 120)_ = 14, *p* < 0.000; $\eta_{\text{p}}^{2}$ = 0.71; PHOB: *F*_(2, 120)_ = 3.7, *p* = 0.030; $\eta_{\text{p}}^{2}$ = 0.27; OTHER: *F*_(2, 120)_ = 5.4, *p* = 0.006, $\eta_{\text{p}}^{2}$ = 0.89]. No differences between groups were found on the remaining dimensions.

The mothers reported higher scores than fathers [GSI: Parent: *F*_(2, 120)_ = 18.7, *p* < 0.000; $\eta_{\text{p}}^{2}$ = 0.82; PST: Parent: *F*_(2, 120)_ = 13.7, *p* < 0.003; $\eta_{\text{p}}^{2}$ = 0.86; PSDI: Parent: *F*_(2, 120)_ = 14, *p* < 0.003 $\eta_{\text{p}}^{2}$ = 0.86] (see Table 2).

**Table 2:** Comparison of psychological profiles between parents of children with ODD, GAD, and TD (separately for mothers and fathers).

	**ODD mothers**	**GAD mothers**	**TD mothers**			**ODD fathers**	**GAD fathers**	**TD fathers**		
	**M; SD**	**M; SD**	**M; SD**	***F***	***p***	**M; SD**	**M; SD**	**M; SD**	***F***	***p***
**SCL-90-R subscales**										
Somatization	0.69; 0.46	0.50; 0.45	0.39; 0.40	(2,120)	0.010^**^	0.41; 0.40	0.30; 0.33	0.32; 0.28	(2, 120)	0.255
Obsessive-Compulsive	0.57; 0.46	0.53; 0.45	0.37; 0.39	(2,120)	0.094	0.45; 0.39	0.55; 0.64	0.36; 0.31	(2, 120)	0.182
Interpersonal Sensitivity	0.54; 0.42	0.44; 0.45	0.33; 0.31	(2,120)	0.058	0.38; 0.33	0.30; 0.32	0.29; 0.31	(2, 120)	0.920
Depression	0.71; 0.48	0.57; 0.38	0.49; 0.48	(2,120)	0.074	0.29; 0.22	0.35; 0.37	0.33; 0.29	(2, 120)	0.822
Anxiety	0.58; 0.39	0.48; 0.40	0.32; 0.35	(2,120)	0.012^*^	0.29; 0.22	0.30; 0.24	0.28; 0.35	(2, 120)	0.981
Hostility	0.67; 0.57	0.43; 0.50	0.35; 0.49	(2,120)	0.019^*^	0.31; 0.36	0.41; 0.74	0.35; 0.43	(2, 120)	0.664
Phobic Anxiety	0.21; 0.28	0.09; 0.20	0.08; 0.02	(2,120)	0.024^*^	0.02; 0.06	0.08; 0.20	0.11; 0.27	(2, 120)	0.096
Paranoid Ideation	0.88; 0.53	0.50; 0.30	0.37; 0.38	(2,120)	0.000^**^	0.48; 0.53	0.42; 0.38	0.46; 0.50	(2, 120)	0.919
Psychoticism	0.31; 0.27	0.21; 0.24	0.15; 0.28	(2,120)	0.029^*^	0.16; 0.25	0.21; 0.31	0.15; 0.21	(2, 120)	0.531
GSI	0.09; 0.20	0.05; 0.03	0.31; 0.30	(2,120)	0.000^**^	0.06; 0.08	0.05; 0.11	0.05; 0.21	(2, 120)	0.291
PST	35; 14	27; 13	20; 14	(2,120)	0.000^**^	20; 15	20; 13	19; 12	(2, 120)	0.900
PSDI	0.71; 0.24	0.74; 0.20	1; 0.05	(2,120)	0.000^**^	0.80; 0.17	0.86; 0.46	0.50; 0.40	(2, 120)	0.000^**^

*SD, standard deviation; SCL-90-R, Symptom Checklist-90-Revised Form; ODD, Oppositional Defiant Disorder; GAD, Generalized Anxiety Disorder; TD, Typical Development; GSI, Global Severity Index; PST, Positive Symptom Total; PSDI, Positive Symptom Distress Index*;

* *p ≤ 0.05*;

** *p ≤ 0.01. The bold values indicate a statistically significant difference with a p < 0.05*.

Overall, parental GSI in SCL-90-R was positively associated with children's problems as referred by parents on the CBCL [GSI in SCL-90-R and Total score on CBCL: *r* = −0.35, *t*_(123)_, *p* < 0.000].

Regarding parental perception of the offspring's psychopathology, on the CBCL total scale, children with ODD received higher scores than TD children. Interestingly, most of the parents of children with ODD reported children's emotional and behavioral problems on the internalizing (74%) and externalizing (64%) scales, respectively.

On the other hand, parents of children with GAD reported a higher number of emotional problems in their children compared to the control group [Total Scale: *F*_(2, 120)_ = 6.9, *p* = 0.001, $\eta_{\text{p}}^{2}$ = 0.8]; Internalizing Scale: *F*_(2, 120)_ = 5, *p* = 0.010, $\eta_{\text{p}}^{2}$ = 0.1; Externalizing Scale: *F*_(2, 120)_ = 3, *p* = 0.050; $\eta_{\text{p}}^{2}$ = 0.4].

On the CBCL 1½−5 subscales, parents of children with GAD and ODD reported higher scores than TD groups on the Anxiety Problems scale [*F*_(2, 120)_ = 10, *p* = 0.001; $\eta_{\text{p}}^{2}$ = 0.8]. Anxiety problems in children with ODD and children with GAD were comparable with no significant difference. In addition, parents of ODD children reported a high number of problems on the Affective and Oppositional Defiant Problems scales [Affective scale [*F*_(2, 120)_ = 7.6, *p* = 0.000; $\eta_{\text{p}}^{2}$ = 0.85; Oppositional Defiant scale: *F*_(2, 120)_ = 4, *p* = 0.000; $\eta_{\text{p}}^{2}$ = 0.8], but not on the Pervasive Developmental problem s[*F*_(2, 120)_ = 2, *p* = 0.090; $\eta_{\text{p}}^{2}$ = 0.5] and ADHD scales [*F*_(2, 120)_ = 0.9, *p* = 0.300; $\eta_{\text{p}}^{2}$ = 8, see Table 3].

**Table 3:** Results of ANOVA analyses of CBCL subscale scores in preschool sample (*n* = 124).

**CBCL 1$\frac{1}{2}$−5**	**ODD group M (SD) *n* = 41**	**GAD group M (SD) *n* = 41**	**TD group M (SD) *n* = 42**	***p***
Internalizing *T*-score	63.31 (10.14)	59.92 (9.03)	51.76 (10.16)	0.000^**^
Internalizing *T* > 63	*N* = 24	*N* = 12	*N* = 1	
Externalizing *T*-score	62.31 (10.14)	52.90 (9.31)	50.38 (10.30)	0.000^**^
Externalizing *T* > 63	*N* = 24	*N* = 5	*N* = 3	
Total problems *T*-score	64.29 (8.75)	57.00 (8.78)	51.95 (9.70)	0.000^**^
Total problems *T* > 63	*N* = 22	*N* = 10	*N* = 3	
Affective problems	62.21 (9.24)	56.68 (7.67)	53.90 (6.43)	0.000^**^
Affective problems *T* > 70	*N* = 11	*N* = 2	*N* = 2	
Anxiety problems	61.22 (10.59)	60.36 (10.22)	53.92 (7.68)	0.001^**^
Anxiety problems *T* > 70	*N* = 9	*N* = 8	*N* = 2	
Pervasive developmental problems	59.65 (7.33)	56.07 (6.89)	55.53 (6.81)	0.090
Pervasive developmental problems *T* > 70	*N* = 10	*N* = 5	*N* = 2	
Attention deficit hyperactivity problems	57.97 (8.83)	54.68 (6.99)	52.85 (4.63)	0.300
Attention deficit hyperactivity problems *T* > 70	*N* = 8	*N* = 3	*N* = 2	
Oppositional defiant problems	64.12 (9.24)	61.58 (8.91)	59.43 (8.42)	0.000^**^
Oppositional defiant problems *T* > 70	*N* = 15	*N* = 4	*N* = 1	

*CBCL, Child Behavior Checklist; SD, Standard Deviation; ODD, Oppositional Defiant Disorder; GAD, Generalized Anxiety Disorder; TD, Typical Development*;

** *p ≤ 0.01. The bold values indicate a statistically significant difference with a p < 0.05*.

## Discussion

While mild and transient manifestations of anxiety and oppositionality are considered indices of child adjustment to stressful life events and individual maturation, a persistent, inappropriate, and pervasive expression of such behaviors are regarded as markers of specific disorders. Thus, early recognition by parents of their child's disorders is fundamental for a prompt and suitable therapeutic intervention. However, parental stress might alter their perception of their offspring's problems (19). To our knowledge, a direct comparison between parental stress and psychopathology between parents of preschool children without a clinical diagnosis and/or a non-comorbid diagnosis of anxiety or ODD has not been conducted so far.

We found that on the SCL-90 R, parents of children with GAD and ODD scored higher on the anxiety and hostility scales compared to parents of children with TD. Presumably, anxiety and hostility mirror parental fears and feelings of powerlessness associated with children's problems and daily negative environmental feedback. On the other hand, parental anxiety and hostility may, in turn, contribute to an increase in children's vulnerability against psychiatric disorders.

Moreover, parental psychological characteristics are differentially related to types of disorder in offspring. Specifically, parents of children with ODD rated high in most of the subscales, which is suggestive of a tendency to not only overestimate a child's disorder, but also the personal problems. Notably, mothers of children with ODD rated high scores on the somatization, depression, anxiety, and paranoid ideation dimensions.

Additionally, high scores on somatization, paranoid, hostility, depression, and anxiety dimensions are associated with an impairment of parental ability to empathize with others' emotions and needs. These manifestations are often associated with a low tuning toward others' perspectives and, presumably, with the offspring's developmental needs and difficulties. This finding is interesting in light of social learning theories, which consider that ODD symptoms are the results of negative reinforcement (41). If the self-concept weakens, the patients are not able to control their emotions and behaviors and the behavioral problems intensify.

Moreover, it seems relevant to note that mothers of children with psychiatric diagnoses rated higher scores than fathers on several dimensions, namely on somatization, depression, anxiety, hostility, and paranoid ideation. These emotional characteristics may interfere with the mother–child relationship and may contribute to the early development of child psychopathology.

Presumably, parents of children with ODD have more problems than parents of children with GAD in elaborating a representation of their own and their children's difficulties.

We found the occurrence of psychiatric disorders on clinical diagnosis was comparable frequently in parents of children with ODD or GAD. Personal psychopathological disorders might have influenced parental perceptions of offspring's emotional and behavioral problems. This explanation fits with the finding on the positive association between the personal global index of stress on the SCL-90-R scale and total children's difficulties on the CBCL scale.

Most of the parents of children with ODD had a representation of their children that was discordant with the clinical diagnosis. Parents of children with ODD overestimated their child's psychopathological difficulties. According to their parents, children with an ODD would not only show the oppositional behaviors, but also behaviors commonly observed in children with emotional disorders.

Parents of children with GAD reported almost exclusively children's emotional difficulties, as was evident on the Internalizing Scale. This result suggested that parents of children with GAD are in tune with their children's actual difficulties.

Presumably, parents of children with ODD have more problems than parents of children with GAD in creating an accurate representation of their own and children's difficulties.

The social impact of the children with ODD could have influenced the parent's overestimation.

It is worth noting that, since the present study was based on correlation analyses, the present findings are not conclusive on any causative relations between variables. Despite this limitation, this study represents one of the few available studies focused on a large sample of preschool children and their families with non-comorbid diagnoses.

Further studies are needed to clarify the interplay between parental coping with life changes and the early emergence of psychiatric disorders in preschool children.

As interpersonal functioning is impaired differently in generalized anxiety and separation anxiety disorders (42), future studies should replicate and extend the investigation of the psychological vulnerability and stress sensitivity in parents of pre-schoolers with separation anxiety disorder.

Overall, the present findings have important implications for clinical practice and the elaboration of research and prevention programs. As the evidence indicates, the understanding of the child's problems and functioning should take into account the daily parental perception of their personal and their offspring's psychological conditions.

The main limitation of the present study is the lack of input on the part of teachers or daycare operators regarding the child's symptoms.

## Data Availability Statement

The raw data supporting the conclusions of this manuscript will be made available by the authors, without undue reservation, to any qualified researcher.

## Ethics Statement

The study was carried out in accordance with the Declaration of Helsinki and approved by the institutional review board of Sapienza–University of Rome. All parents gave a written informed consent to participate in this study.

## Author Contributions

FM, FG, and CS contributed to the study design, supervised and managed data collection, performed statistical analyses, were involved in the data interpretation and the conceptualization of the paper, wrote the first draft of the manuscript, critically revised the manuscript, and approved the final version of the manuscript submitted.

### Conflict of Interest

The authors declare that the research was conducted in the absence of any commercial or financial relationships that could be construed as a potential conflict of interest.
